# Optimization of Formulation Parameters for Reduced-Fat Katima with Improved Nutritional Quality Using Response Surface Methodology and Evaluation of Quality Characteristics

**DOI:** 10.3390/foods15152684

**Published:** 2026-07-30

**Authors:** Huiling Huang, Zhichao Yang, Tiemier Kanjiguli, Zhelong Kang, Lin Ye, Xujie Hou, Yiming Jia, Shenghuizi Chen, Ying Huang

**Affiliations:** School of Food Science and Engineering, Tarim University, Alar 843300, China

**Keywords:** Katima, sensory evaluation, response surface, process optimization

## Abstract

This work optimized the formulation of the traditional Kyrgyz layered pastry Katima to lower its high butter-derived lipid content. High-gluten flour served as the base raw material, with butter, yeast, salt, egg liquid, and raisins as auxiliary ingredients. Single-factor trials combined with Box–Behnken response surface methodology (RSM) were carried out, with overall sensory score as the primary evaluation index to screen critical formulation variables: butter, yeast, and salt exerted distinct linear and interactive effects on product acceptability. The validated optimal formula contained 12.67 g butter, 0.78 g instant active dry yeast, and 0.56 g edible salt per 100 g flour, yielding a mean sensory score of 95.03 and a 37.21% fat reduction relative to full-fat traditional Katima. Significant intergroup differences (*p* < 0.05) were observed in texture, surface color, and physicochemical profiles. Reduced-fat Katima exhibited elevated moisture, hardness, and chewiness, alongside markedly lower fat, peroxide and acid values, reflecting enhanced lipid oxidative stability. This study provides standardized technical parameters and a feasible upgrading strategy for industrialized production of healthy Xinjiang ethnic cereal pastries.

## 1. Introduction

Katima is a characteristic layered cereal pastry of China’s Kyrgyz ethnic group. Prepared via spiral rolling and low-temperature baking with abundant clarified butter, traditional Katima features a crispy multi-layered texture and natural whole-grain nutrition rich in dietary fiber, B vitamins, and minerals [1]. Nevertheless, its high butter dosage (21%) leads to excessive total fat, conflicting with the reduced-fat, whole-grain dietary guidance of the Chinese Dietary Guidelines (2022). Current Katima studies merely optimize baking and fermentation parameters on full-fat formulas, with very limited research targeting fat-reformulation strategies. Additionally, ethnic cereal pastry research overall lags far behind investigations on Central Asian fermented dairy products. The empirical, manual traditional production mode also causes unstable product quality in industrialized batch processing. Against these gaps, this study optimizes formulation parameters for Katima with Improved Nutritional Quality to balance reduced-fat properties, sensory quality, and standardized production.

Dairy butter serves as the key lamination and flavoring material for layered pastries like Katima but is rich in saturated fatty acids. Long-term overconsumption of high-butter baked goods raises risks of obesity and cardiovascular diseases, making reduced-fat reformulation of ethnic butter-containing pastries an urgent demand. However, simple butter reduction severely damages pastry texture, a core technical barrier for reduced-fat pastry production. As Huang et al. [2] summarized, milk fat crystals with stable β’ polymorphs lubricate gluten chains, stabilize gas pores, and plasticize gluten networks during dough processing. Sharp butter depletion eliminates lipid isolation layers between gluten molecules, strengthens protein crosslinking, and greatly increases hardness and chewiness while lowering springiness; similar texture deterioration caused by fat cutting has been widely reported in bread, biscuits, and laminated pastry studies.

Various fat replacers (oleogels, protein gels, carbohydrate mimetics, amylose-lipid complexes (ALCs)) have been developed to offset such quality loss. Lee et al. [3] proved corn starch-stearic acid ALCs could lower calorie intake and suppress starch retrogradation at a 50% shortening substitution rate; yet excessive ALCs hinder dough aeration and shrink product volume, restricting their use in layered pastries dependent on butter for sheet separation. Kathryn et al. [4] further compared common fat substitutes: oleogels reduce saturated fatty acids but retain high total lipid, while carbohydrate-based mimics deliver low calories but fail to replicate butter’s unique creamy taste and lamination performance. Accordingly, most fat-reduction studies target Western bread and cakes, with rare research focusing on Xinjiang traditional layered pastries such as Katima.

Response surface methodology (RSM) is a mainstream multivariate optimization tool for reduced-fat bakery products. It establishes quadratic regression models to quantify interactive effects of multiple formula variables on sensory and textural properties, overcoming the limitation of single-factor tests that only reflect linear ingredient influences and accelerating the screening of balanced reduced-fat formulas [5,6]. Current domestic Katima research only applies RSM to optimize baking and fermentation parameters of full-fat prototypes, without taking butter reduction as the core optimization objective, nor systematically regulating butter, yeast, and salt dosages to balance lipid content and edible quality. Different from most existing studies that add exogenous fat replacers to relieve texture defects, this work optimizes the original Katima formula merely by adjusting butter dosage via RSM, avoiding extra additives and fitting the simple industrial processing demands of ethnic pastries. This study therefore fills the research gap of targeted reduced-fat reformulation for Kyrgyz Katima.

## 2. Materials and Methods

### 2.1. Materials and Instruments

Eggs, raisins, and white granulated sugar were purchased from the campus market of Tarim University (Alar, Xinjiang, China). Unsalted butter (82% milk fat, 16.5% water, and 1.5% non-fat milk solids) was supplied by Fonterra Group (Auckland, New Zealand). High-gluten wheat flour with a crude protein content of 13.2 g/100 g (dry basis) was obtained from Xingtai Jinshahe Flour Industry Co., Ltd. (Xingtai, Hebei, China). Instant active dry yeast with viable cell counts ≥ 2.0 × 10 CFU/g and moisture content ≤ 6.0% was provided by Xinjiang Mali Food Co., Ltd. (Urumqi, Xinjiang, China). Refined edible salt was sourced from Turpan Lianda Salt Co., Ltd. (Turpan, Xinjiang, China). All experimental raw materials were of food grade and used directly without further purification.

All raw materials and test samples were weighed using an analytical balance (Model MT620, Mettler Toledo, Greifensee, Switzerland; maximum weighing capacity: 620 g, readability: 0.01 g). Dough fermentation was performed in a constant-temperature incubator (Model M1712, Beijing Haifuda Technology Co., Ltd., Beijing, China; temperature control range: 5–60 °C, temperature precision: ±0.1 °C). Katima sample baking was completed using an electric baking oven (Model MD-1052, Midea Group Co., Ltd., Foshan, Guangdong, China; adjustable temperature range: 30–230 °C, maximum rated power: 1600 W). No analytical software was used in this section of the experiment.

### 2.2. Experimental Methods

#### 2.2.1. Katima’s Process Flowchart

**Figure 1:**
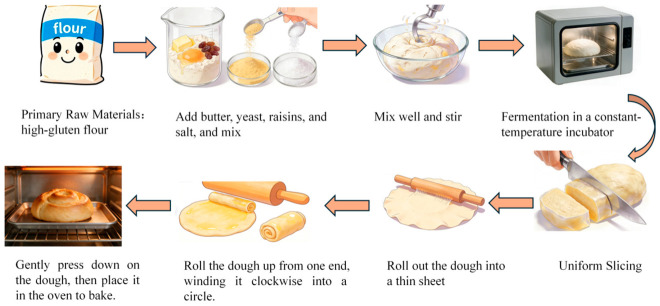
Schematic flowchart for the production process of Katima, a traditional pastry. The sequential orange solid arrows represent the forward technological operation sequence from raw material preparation to finished product baking; the curved orange arrow indicates the cyclic transfer of fermented dough for subsequent shaping procedures. The eight unit operations in order are: high-gluten flour preparation, auxiliary ingredient addition and premixing, dough kneading, constant-temperature fermentation, uniform dough slicing, dough sheeting, clockwise rolling molding, and oven baking.

The Katima manufacturing process is shown in Figure 1.

#### 2.2.2. Preparation Steps

Detailed steps of the Katima production process is shown in Table 1.

### 2.3. Single-Factor Experimental Design

In this study, a sensory evaluation protocol was adopted under fixed ingredient dosage conditions to investigate the effects of varying ingredient additions on the sensory quality of the final product. The five variables examined included the dosages of egg liquid, butter, yeast, raisins, and salt. In accordance with the single-factor experimental principle, each variable was evaluated at five fixed levels: egg liquid (0, 15, 25, 35, and 50 g), butter (5, 10, 15, 20, and 25 g), yeast (0.2, 0.4, 0.6, 0.8, and 1.0 g), raisins (0, 4, 8, 12, and 16 g), and salt (0, 0.2, 0.4, 0.6, and 0.8 g). After the completion of each single-factor test, sensory evaluation was performed to analyze the impacts of different ingredient dosages on product quality.

All factor ranges and five gradient levels of single-factor experiments were determined based on preliminary exploratory pre-experiments. Five gradient levels were adopted to balance experimental efficiency and the ability to identify the changing trend of evaluation indexes; the parameter range was screened to exclude intervals with poor product quality observed in pre-tests, ensuring that the optimal processing condition fell within the test range. For each experimental level, three independent production batches of Katima were prepared on separate days (*n* = 3), instead of analytical replicates of a single sample, to evaluate the total experimental variation of the whole processing procedure.

### 2.4. Response Surface Methodology for Process Formula Optimization

Following the acquisition of single-factor experimental data, response surface methodology (RSM) trials were carried out. Egg liquid (20 g) and raisins (8 g) were set as constant variables, whereas butter, yeast, and salt were designated as the three independent experimental factors. A three-factor, three-level Box–Behnken design was adopted for this study, and the complete experimental matrix is listed in Table 2. The optimized processing parameters predicted by the model were further validated via confirmatory experiments. Quadratic polynomial regression models were constructed to quantify the correlations between the three formulation variables and each response indicator. All regression models fulfilled the fundamental statistical prerequisites for RSM analysis, including normally distributed residuals, homoscedasticity, and independent random errors. The coefficient of determination (R^2^), adjusted (R^2^) and lack-of-fit *p*-values were computed to assess the goodness-of-fit of each model, and residual diagnostic plots were generated to validate model reliability. The 95% confidence intervals for the predicted optimal formulation parameters were calculated and compiled in the combined analysis of variance (ANOVA) table.

(1) Experimental design type: A three-factor, three-level Box-Behnken design (BBD) was adopted for response surface modeling. (2) Software information: All experimental design, quadratic regression model fitting, significance analysis, and multi-objective optimization were conducted using Design-Expert Version 13 software. Default significance thresholds were set as *p* < 0.05 for significant terms and *p* < 0.01 for highly significant terms. (3) Coded quadratic regression equations: The full coded regression equation taking sensory score as the response value was supplemented in the manuscript. (4) Optimization criteria: Single-objective optimization was carried out targeting the maximum comprehensive sensory score of reduced-fat Katima; the range of each factor was limited to the test intervals obtained from single-factor experiments. (5) Desirability function: The desirability approach built into Design-Expert 13 was applied. The desirability value was set to range from 0 (unacceptable) to 1 (optimal), and the software calculated the overall desirability to screen the optimal formula combination.

### 2.5. Model Verification

The conventional Katima production process involves the use of 200 g flour, 1.7 g salt, and 42 g butter, undergoing fermentation, rolling, and baking. The optimal formula for reduced-fat nutritional Katima, determined through response surface methodology, was validated through three replicates.

### 2.6. Nutritional Composition Analysis

According to national standard analytical methods, the contents of fat, protein, and other components were determined in both conventional and optimized Katima samples.

### 2.7. Texture Determination

Texture profile analysis was conducted on Katima specimens cut into rectangular blocks of 30 mm × 50 mm × 50 mm, following the analytical protocol reported by Ropciuc et al. [7]. Measurements were performed using a texture analyzer (Model TMS-PILOT, Food Technology Corporation (FTC), Sterling, VA, USA) fitted with a flat-bottom cylindrical P36 R probe. The pre-test speed was set to 5 mm/s, whereas the test and post-test speeds were both fixed at 1 mm/s, with a compression deformation rate of 50%. Four textural indices, namely hardness, springiness, adhesion, and chewiness, cohesiveness, were quantified. All tests were performed in triplicate.

### 2.8. Crumb Density Determination

Three Katima specimens were randomly selected for crumb density measurement. The length, width, and thickness of each sample were measured with a vernier caliper to calculate individual volume, while an electronic balance was used to record the mass of each piece. crumb density was then computed via the corresponding formula, and the average value of three replicates was recorded as the final experimental result. The calculation formula for crumb density is shown below.

ρ = m/V(1)

Here, ρ is the crumb density (g/cm^3^), m is the sample mass (g), and V is the sample volume (cm^3^).

### 2.9. Colour Difference Analysis

The surface color of Katima specimens was determined using a CR-400 colorimeter (Konica Minolta Corporation, Tokyo, Japan). The L* value represents lightness, with higher values indicating a lighter surface appearance; the a* value reflects the red–green chromatic axis, where larger positive values signify a redder color; the b* value denotes the yellow–blue chromatic axis, and higher positive values correspond to a yellower color. The total color difference (ΔE) was further calculated from the measured L*, a*, and b* values in accordance with the method described by [8].

### 2.10. Physical and Chemical Parameter Analysis

Moisture content was tested via the direct drying method specified in the Chinese National Food Safety Standard GB 5009.3–2016, which aligns with ISO 1442:1997 [9]. Acid value was determined according to GB 5009.229–2016, consistent with ISO 660:2020 [10]. Peroxide value was quantified following the protocol of GB 5009.227–2016, equivalent to ISO 3960:2017 [11]. Fat content was measured by Soxhlet extraction as outlined in GB 5009.6–2016, matching ISO 11085:2015 [12].

### 2.11. Sensory Evaluation

A total of 20 food science undergraduates aged 18–24 years were recruited as sensory panelists. Exclusion criteria included taste or smell dysfunction, oral lesions, food allergies to Katima raw materials, smoking habits, and recent consumption of medications that interfere with taste perception. All participants underwent standardized calibration training prior to formal testing: reference samples of traditional full-fat Katima were used to unify scoring criteria for four sensory attributes, and repeated practice was conducted until the inter-panel scoring consistency exceeded 85%. All samples were blindly coded and evaluated in two separate replicate sessions; two-way analysis of variance (ANOVA) confirmed no significant differences between replicates (*p* > 0.05), indicating stable sensory reproducibility. All volunteers signed informed consent forms before testing.

All samples were labeled with random three-digit blind codes and presented to panelists in a fully randomized order. Sensory evaluation was conducted in an independent, quiet sensory room under uniform soft white light and constant room temperature (25 °C). Panelists rinsed their mouths with purified water and waited for 5 min between consecutive samples to eliminate cross-flavor interference.

Currently, there is no special national sensory evaluation standard for Katima. The scoring criteria adopted in this study were established by referring to the existing sensory evaluation standards for traditional high-oil baked pastries in Xinjiang. Four evaluation indicators were set, including colour, texture, taste, and smell. Among them, taste was assigned a score of 40 points, while colour, texture, and smell each accounted for 20 points. Taste was given a higher weight because it is the most concerned quality characteristic of Katima for local consumers. The above scoring scheme was discussed and confirmed by professional food sensory evaluation teachers in our college.

Prior to formal sensory testing, all panelists received 5 rounds of standardized training using reference Katima samples to unify scoring criteria; assessors with intra-individual scoring errors over 10% were excluded. Cronbach’s alpha was computed to assess the internal consistency of sensory attribute scores. The Cronbach’s α values for appearance, texture, aroma, and taste attributes were 0.88, 0.93, 0.86, and 0.91, respectively, all exceeding the acceptable threshold of 0.80, indicating high internal reliability of the sensory panel. Two panel performance indices were further calculated: intra-assessor repeatability and sample discrimination F-statistic. The low intra-individual error and significant inter-sample F-values confirmed that the panel possessed stable, consistent discrimination capacity for all experimental pastry samples, ensuring good reproducibility of sensory results.

Sensory testing was performed in three independent batches on separate days. The coefficient of variation (CV) of sensory scores was calculated to assess intra-panel repeatability, and low CV values confirmed consistent scoring performance across all panel members. A small pilot test was carried out before formal sensory trials to revise vague descriptive wording within the scoring rubric. It is objectively acknowledged that this self-developed scoring system has certain limitations: weight distribution was determined according to the preference of local consumers instead of universal international sensory standards. Future research will recruit more diverse consumer groups and fully trained professional sensory panels to optimize and validate the present evaluation framework. The complete scoring criteria are summarized in Table 3.

### 2.12. Data Statistics and Analysis

All experiments and analytical measurements were conducted in triplicate. Origin 2021 (OriginLab Corporation, Northampton, MA, USA) was used for data visualization and graph plotting, whereas Design-Expert 13 (Stat-Ease, Inc., Minneapolis, MN, USA) was employed for all response surface methodology (RSM) statistical calculations. A Box-Behnken design (BBD) was selected for RSM optimization, with a total of 17 experimental runs, including 3 central replicate points. A quadratic polynomial regression model was constructed based on the effective dosage ranges obtained from single-factor trials. Analysis of variance (ANOVA) was performed to examine the statistical significance of the overall regression model, linear terms, quadratic terms, and pairwise interaction terms. Multi-objective optimization via the desirability function embedded in Design-Expert 13 was implemented to determine the optimal formulation parameters.

All statistical analyses were performed using IBM SPSS Statistics 26 software (IBM Corporation, Chicago, IL, USA). Prior to one-way analysis of variance (one-way ANOVA), normality and homogeneity of variance tests were conducted to verify the prerequisites for variance analysis., the Shapiro–Wilk test and Levene’s test were performed sequentially to verify data normality and variance homogeneity, respectively. Only datasets that satisfied both prerequisites (*p* > 0.05) proceeded to standard one-way ANOVA. All measurement data in this study passed the Shapiro-Wilk normality test and Levene’s homogeneity of variance test (*p* > 0.05), meeting the basic assumptions of one-way ANOVA. The corrected LSD post hoc test was adopted for pairwise comparisons.

The LSD test was selected due to the study’s simple two-group design (optimized reduced-fat Katima and conventional full-fat Katima). For pairwise comparisons limited to only two groups, there is no accumulation of familywise Type I error, which eliminates the well-known drawback of LSD in multi-group comparison scenarios. Compared with stricter multi-group correction methods such as Tukey’s HSD, LSD retains higher statistical sensitivity to detect subtle differences between the two pastry formulations without elevating the false positive probability under the current experimental setup. For future trials containing three or more treatment groups, Tukey’s HSD test will be prioritized to strictly control Type I error.

All quantitative data were expressed as the mean ± standard deviation, and differences with *p* < 0.05 were considered statistically significant.

## 3. Results and Discussion

### 3.1. Single Factor Experiment

#### 3.1.1. The Effect of Adding Eggs on the Quality of Katima

**Figure 2:**
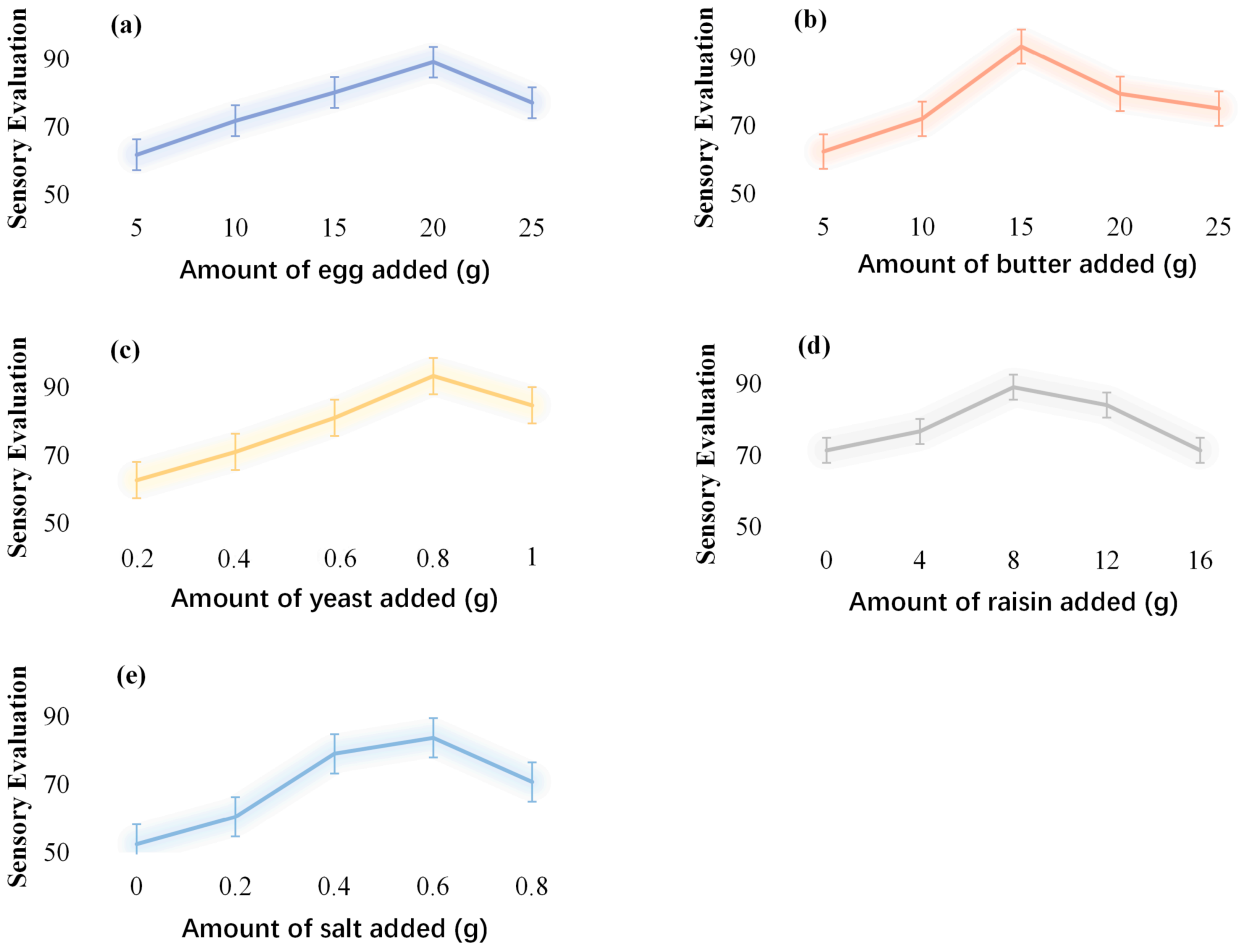
The factors that affect the sensory score of Katima include egg (**a**), butter (**b**), yeast (**c**), raisins (**d**), and salt (**e**). All determinations were performed in triplicate (*n* = 3). Vertical error bars represent standard deviation.

Single-factor studies were carried out individually for egg liquid, butter, yeast, raisins, and salt to explore the independent effects of each ingredient on the sensory attributes and textural properties of Katima, and the corresponding outcomes are illustrated in Figure 2a. Incorporating egg liquid into the dough improves softness and facilitates the development of a porous structure, which yields a crisp texture in finished Katima. As depicted in Figure 2a, sensory scores rose first and declined subsequently with increasing egg dosage, reaching a maximum value of 89 points at an egg liquid addition level of 20 g.

Eggs exert multifunctional roles throughout Katima processing, acting as binders, foaming agents, and substrates for the Maillard reaction. Egg albumin accelerates disulfide cross-linking between gluten proteins to strengthen the dough network, which improves structural integrity and enhances the plumpness, surface color, and flavor of baked products [13]. Nevertheless, egg liquid dosages exceeding 20 g introduce excessive protein, raising dough viscosity and triggering irregular shaping of pastries; an overabundance of egg ingredients generates an overly tender, collapsible texture; meanwhile [14], jointly impairing the overall sensory acceptability of samples.

#### 3.1.2. The Effect of Adding Butter on the Quality of Katima

As shown in Figure 2b, sensory scores of Katima exhibited a fluctuating trend with increasing butter addition, peaking at 93.33 points when 15 g of butter was incorporated. Butter fulfills three critical functions in Katima preparation: enhancing flakiness, providing lubrication, and imparting a characteristic flavor. At the optimal dosage, butter improves dough extensibility and rollability, forming a more porous, layered structure and enriching the product’s flavor. However, when the amount of butter exceeds 15 g, it can result in poor shape stability, a greasy texture, and excessively high fat content, which can mask the flavors of the other ingredients [15]. Ultimately, this reduces sensory acceptability. This trend aligns with the observations reported by Srikanlaya et al. [16] in their study on butter dosage modifying bread textural and rheological properties.

In the single-factor experiment, five butter addition levels were set at 5, 10, 15, 20, and 25 g. Sensory results showed that the 15 g butter group had the best overall sensory profile; although 15–25 g of butter can enhance the softness and milky aroma of the pastries, excessively high fat content contradicts the core objective of this study, which is reduced-fat reformulation. Therefore, the optimization range was narrowed to 10–15 g to conduct a response surface experiment; the response surface center point of 12.5 g is the midpoint of this range and is the standard value for the Box-Behnken design, which does not need to coincide with the single-factor gradient.

#### 3.1.3. The Effect of Adding Yeast on the Quality of Katima

Yeast dosage acts as a vital processing variable for Katima manufacture. Insufficient yeast addition limits gas generation throughout fermentation and leads to incomplete dough proofing. Such dough develops compact, tiny internal pores, yielding a dense texture and an undesirable mouthfeel [17]. In contrast, excessive yeast triggers irregular product morphology, surface blistering, and cracking. Over-fermentation accumulates abundant gas, which loosens the gluten matrix and causes finished pastries to collapse. Moreover, alkaline metabolites from overactive yeast amplify off-notes of alkalinity and disrupt the balanced flavor profile of Katima [18].

As illustrated in Figure 2c, sensory scores of Katima rose first and then declined as yeast dosage increased, reaching the maximum value at 0.8 g. At this optimal concentration, Katima displayed moderate expansion volume, uniform delicate texture, and satisfactory palatability. Once yeast addition surpassed 0.8 g, surplus yeast cells depleted dough carbohydrates and generated excessive organic acids and ethanol, bringing about noticeable sour notes and a subsequent drop in sensory scores.

#### 3.1.4. The Effect of Adding Raisins on the Quality of Katima

Raisins incorporated into Katima enrich its flavor and textural properties, endowing the pastry with a balanced sweet–sour taste and layered mouthfeel. As illustrated in Figure 2d, sensory scores increased gradually with elevated raisin dosage before declining, reaching a peak value of 89 points at an addition level of 8 g. Raisin dosages above 8 g dehydrated the dough matrix, creating a coarse texture and overwhelming the delicate flavor balance of other raw materials. Furthermore, excess raisins lead to uneven heat distribution during baking and irregular pastry shaping, which reduces overall sensory scores [19]. Owing to its relatively minor influence on comprehensive sensory quality compared with butter, yeast, and salt, raisin dosage was fixed at 8 g and excluded as an optimization factor in the subsequent response surface design.

#### 3.1.5. The Effect of Adding Salt on the Quality of Katima

Salt fulfills two core functions during Katima processing: flavor modulation and gluten network reinforcement. Moderate salt addition enriches flavor complexity and improves dough elasticity and structural stability, yielding uniformly shaped finished pastries [20]. As illustrated in Figure 2e, sensory scores rose gradually with increasing salt dosage and then declined, reaching a maximum value of 83.67 at a salt addition of 0.6 g. Excess salt generates overwhelming salty notes that suppress the inherent flavors of other raw materials and simultaneously suppress the metabolic activity of yeast during fermentation [21]. This insufficient fermentation yields dense, rigid dough and weakens overall sensory acceptability, which accounts for the downward trend in sensory scores beyond the optimal salt level.

As shown by the results of the one-factor analysis, compared with butter and yeast, eggs and salt have a significantly weaker effect on the overall sensory quality of the product. Therefore, in the response surface design, the amounts of eggs and raisins were both set as fixed parameters and were not adjusted as optimization variables.

Data processing fitting the regression equation:

Y = 95.42 + 0.8875A − 0.5375B − 1.63C + 2.40AB − 1.03AC + 2.57BC − 6.19A^2^ − 4.34B^2^ − 5.26C^2^

The variance analysis and fitting analysis of the model are shown in Table 4.

### 3.2. Analysis of Response Surface Experiment Results

#### 3.2.1. Response Surface Methodology Model Fitting

**Figure 3:**
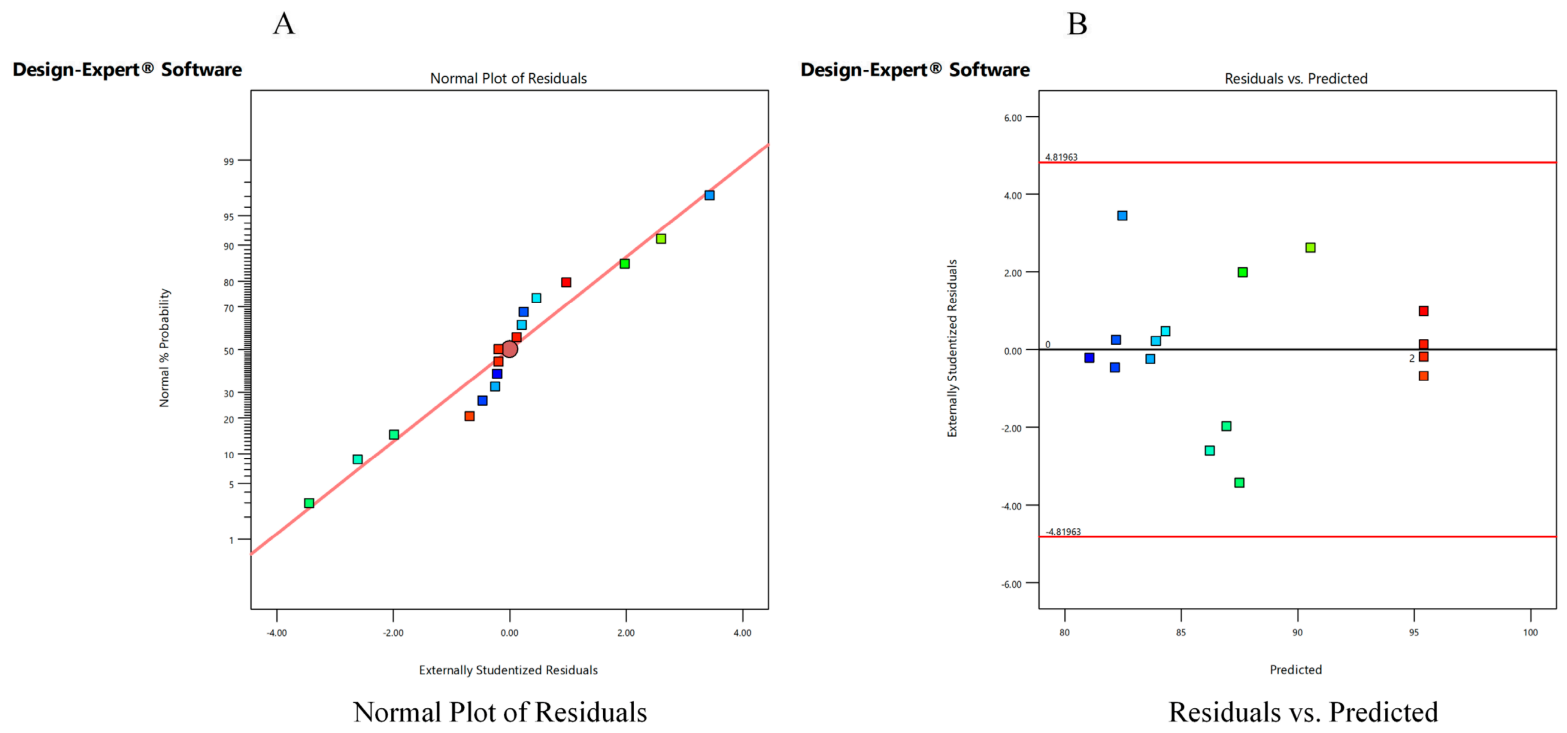
Diagnostic residual plots of the quadratic regression model for the comprehensive sensory score. ((**A**) Normal probability plot of externally studentized residuals; (**B**) Plot of externally studentized residuals versus predicted values.). Each colored square represents an independent experimental run in the Box-Behnken design, where blue, cyan, green, yellow, orange, and red squares correspond to different combinations of independent variable levels in the experimental matrix. The upper and lower red horizontal lines in (**B**) denote the ±4.81963 threshold limits for externally studentized residuals. All residual points are uniformly distributed within the limit bands without obvious outliers, demonstrating that the model satisfies the assumptions of normality and homoscedasticity.

The response surface design was used to optimize the production process of Katima based on the single-factor experiment, and the optimal process parameters were determined by sensory evaluation. The response surface experimental results are shown in Table 4. As shown in the Figure 3, Residual analysis confirmed that the model met the prerequisites of normal distribution and homoscedasticity of residuals.

ANOVA results revealed that the regression model was highly significant, with an F-value of 122.23 (*p* < 0.0001). The coefficient of determination (R^2^) and adjusted (R^2^) were 0.9937 and 0.9855, respectively. The gap between adjusted (R^2^) and predicted (R^2^) was smaller than 0.2, indicating good consistency and the absence of obvious overfitting, which verified the outstanding fitting performance and reliability of the model. As presented in Table 5, the lack-of-fit F-value was 6.11 (*p* = 0.0564), demonstrating negligible model error and satisfactory rationality for predicting the optimal formulation. Single-factor analysis showed that the linear terms of butter dosage (A) and salt dosage (C) exerted significant effects on sensory scores, whereas yeast dosage (B) had no statistically significant influence. All interaction terms (AB, AC, BC) reached significant or extremely significant levels, and all quadratic terms (A^2^), (B^2^), (C^2^) exhibited highly significant nonlinear effects. The comprehensive influence sequence of the three variables followed the order: salt addition > butter addition > yeast addition. The quadratic polynomial model for the sensory evaluation score was extremely significant (*p* < 0.0001), while the lack-of-fit term was non-significant (*p* = 0.0564 > 0.05), further confirming the model’s strong fit to the experimental data. The coefficient of variation (CV) was 0.7514%, representing minor experimental error and excellent test repeatability. The adequate precision value was 28.2864, far above the critical threshold of 4. This result confirmed a favorable signal-to-noise ratio, proving the model could reliably traverse the entire experimental design space for optimization.

#### 3.2.2. Response Surface Optimization for the Processing Technology Formula of Reduced-Fat Katima

The response surface was drawn by Design-Expert 13 to analyze the effects of the amount of butter, salt, and yeast and the interaction of the factors on the sensory evaluation.

((A) Interaction between butter addition and yeast addition; (B) Interaction between butter addition and salt addition; (C) Interaction between yeast addition and salt addition. The vertical axis represents the comprehensive sensory score of Katima, while the horizontal axes correspond to the dosage levels of the two selected raw materials. The curved surface and contour lines reflect the variation trend of sensory scores under different combined dosages of the two factors, and the peak position of each surface corresponds to the optimal matching range of the two variables for maximum sensory acceptability.)

**Figure 4:**
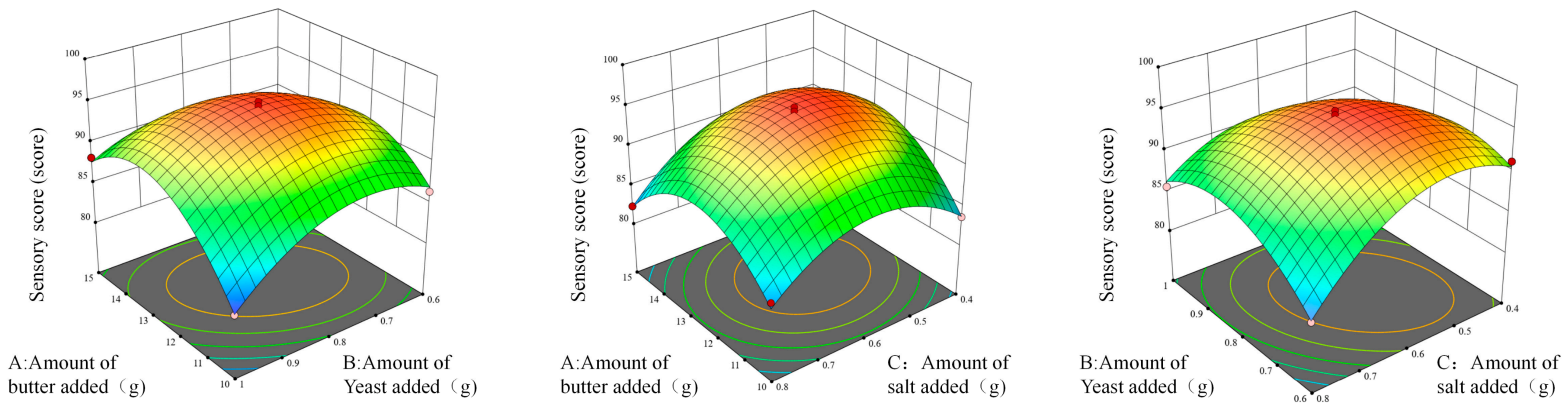
Response surface plots illustrating the interactive effects of two formulation variables on the overall sensory score of reduced-fat Katima.

Figure 4 visualizes the pairwise interactive effects of the three formulation variables (butter, yeast, and salt) on sensory scores via three sets of response surface. Subplot A illustrates the interaction between butter and yeast dosage. The steep gradient and densely packed flat elliptical contour lines of the corresponding response surface verify that the interaction between butter and yeast is highly significant and has a strong impact on overall sensory scores. Subplot B reflects the interaction between butter and salt dosage. Its gently sloped surface and near-elliptical contour lines indicate a relatively weak interactive effect that only slightly alters sensory performance. Subplot C depicts the interaction between yeast and salt dosage. This surface has a steeper gradient and clearer elliptical contour distribution than Subplot B, demonstrating a prominent significant interaction between yeast and salt that substantially changes sensory evaluation results. Based on the ANOVA data compiled in the revised combined Table 4, the significance of each independent variable (butter, yeast, salt) for each response indicator was analyzed according to *p*-values. Butter exerted an extremely significant influence on fat content, hardness, and adhesiveness (*p* < 0.01), making it the dominant factor. Yeast significantly regulated springiness and sensory scores (*p* < 0.05), whereas salt mainly affected cohesiveness with a weaker overall effect. Significant two-way interactions between butter–yeast and butter–salt were also detected for chewiness and comprehensive sensory performance. All quadratic regression equations achieved high fitting precision, with (R^2^ > 0.94) for every response index and non-significant lack-of-fit values, which validated the reliability of the constructed mathematical models. Multi-objective optimization implemented in Design-Expert 13 yielded the optimal formulation for reduced-fat Katima: 12.67 g butter, 0.78 g yeast, and 0.56 g salt. The model predicted a sensory score of 95.63 for Katima prepared with this optimized formula.

#### 3.2.3. Validate the Optimal Machining Process Formula

Optimal processing parameters were identified via the response surface analysis platform Design-Expert 13. Considering practical production operability, three parallel validation trials were performed using the optimized formulation: 12.67 g butter, 0.78 g yeast, and 0.56 g salt. The sensory scores obtained from the three replicate tests were 94.6, 95.3, and 95.2, respectively. The average sensory score of the reduced-fat Katima manufactured with this formula reached 95.03 ± 0.39 (*n* = 3). The relative error was only 0.99% compared with the model-predicted theoretical value of 95.63. The 95% confidence interval for the predicted sensory score (95.63) was calculated to be [94.25, 96.99], and the experimental average score of 95.03 fully falls within this confidence range. The close agreement between experimental measurements and model predictions confirms that the established regression model possesses reliable predictive capacity, and RSM is a feasible tool for optimizing the formulation of reduced-fat Katima.

### 3.3. Texture Characteristics

Textural properties of reduced-fat Katima and traditional Katima are summarized in Table 6. Compared with traditional Katima, the optimized reduced-fat sample showed significantly higher hardness, springiness, cohesiveness, and chewiness (*p* < 0.05), whereas its adhesiveness decreased markedly. Butter serves as a lubricant and tenderizer in baked dough systems. Lower butter addition reduces the proportion of the oil phase in the dough matrix, eliminating lipid lubrication between gluten filaments and pastry layers; this directly elevates the firmness, hardness, and chewiness of finished products. The textural disparities between reduced-fat Katima and traditional full-fat counterparts observed herein align with core mechanistic trends documented in recent reduced-fat laminated pastry research; yet, nuanced differences emerge when compared to Western croissant and bread reformulation studies. Consistent with Esmaeilinezhad Atefeh et al. (2025) [22], reduced lipid dosage eliminates continuous lipid lubricating films interspersed within gluten networks, intensifying protein cross-linking and thereby elevating hardness and chewiness. However, the magnitude of texture change in this work is milder than reported for zero-butter wheat bread (Jia et al., 2022) [23], a discrepancy attributable to the retained moderate butter dosage (12.67 g/100 g flour) rather than complete fat substitution via exogenous gel-based fat mimetics adopted in most Western bakery trials.

### 3.4. Crumb Density Analysis

Crumb density values of reduced-fat Katima and traditional Katima are listed in Table 6.

Higher crumb density in reduced-fat Katima mirrors the findings of Chin et al. (2010) [24], who verified that milk fat promotes thermal bubble expansion during baking to form porous internal crumb structures. Quantitative comparison with Angioloni & Collar (2010) [25] reveals that the crumb density increment (0.0372 g/cm^3^) in this experiment is far lower than the density shift observed in high-fiber low-calorie bread without dairy fat, indicating that residual butter still exerts moderate foaming and plasticizing effects. Unlike industrial puff pastry studies relying on oleogel-based fat replacers to maintain volume, this work achieves acceptable pastry expansion without functional lipid substitutes, yet the elevated compactness still imposes clear limitations on product mouthfeel. From an industrial production perspective, the higher crumb density may restrict application scenarios targeting ultra-light layered pastry products, a constraint that should not be overlooked in technical translation.

### 3.5. Color Difference Analysis

L*, a* and b* are standard parameters of the CIE Lab color system widely used to characterize food surface color. L* stands for lightness, with larger values representing a brighter appearance; positive a* values indicate a red tone; positive b* values reflect a yellow tone [26]. Color differences were observed between reduced-fat and traditional Katima (*p* < 0.05), as listed in Table 6. The reduced-fat sample had a lower L* value (58.51 vs. 68.13) (darker surface), higher a* value (8.98 vs. 0.65) (stronger red tone) and higher b* value (34.94 vs. 27.69) (deeper yellow tone). The decrease of butter proportion raises the relative content of flour, protein and carbohydrate in dough. Without sufficient oil to isolate solid raw materials, these hydrophilic components contact water more sufficiently, accelerating Maillard browning reactions during baking. Browning reaction produces melanoidin substances, darkening the product surface and strengthening red and yellow hues.

The total color difference ΔE between two groups was calculated as 14.65. Generally, ΔE > 3 means a distinguishable color difference to the naked eye, and ΔE > 12 represents a prominent color divergence. The ΔE value of 14.65 in this study proved that butter dosage exerted an obvious influence on Katima surface color.

### 3.6. Physical and Chemical Test

Physicochemical indexes corresponding to Table 6. As shown in Table 6 reducing the butter dosage caused significant differences in multiple physicochemical indicators of Katima. The moisture content of reduced-fat Katima (9.627%) was remarkably higher than that of traditional Katima (7.95%). Butter exhibits inherent hydrophobic characteristics, which readily coat flour particles and gluten chains during dough mixing, as noted by Yazar et al. [27]. This hinders the binding of gluten hydrophilic residues to free water, thereby inhibiting the water-binding capacity of the gluten network and ultimately reducing the dough’s overall water-holding capacity. Once butter addition is lowered, this lipid shielding layer is largely removed, allowing gluten proteins to fully hydrate without physical obstruction and effectively enhancing the water-holding capacity of the pastry matrix, which explains the higher residual moisture detected in the optimized reduced-fat Katima.

The fat content of the optimized Katima formulation was reduced to 10.77%, substantially lower than the 17.21% measured for the traditional full-fat counterpart, a direct outcome of the lowered butter addition level. Correspondingly, the optimized reduced-fat sample also exhibited markedly decreased peroxide value (275.97 meq O_2_/kg) and acid value (0.111 mgKOH/g), compared to 316.34 meq O_2_/kg and 0.132 mgKOH/g recorded in conventional Katima. Fundamentally, lipid auto-oxidation and lipolytic hydrolysis reactions are dependent on the abundance of lipid substrates within the baked matrix; diminishing total fat dosage directly cuts the pool of substrates available for rancidity reactions, thus slowing lipid degradation and yielding mild oxidative indicators, which is a well-established universal trend across low-moisture baked matrices, as verified by Gumus et al. [28]. Their cracker trials with gradient fat concentrations demonstrated a clear positive correlation between total lipid content and the accumulation of primary and secondary oxidation products, including lipid hydroperoxides and hexanal; formulations with higher fat levels presented shorter oxidation lag periods and faster rancidity development, while reduced-fat matrices significantly retarded oxidative deterioration due to limited lipid substrates.

Consistent with the substrate-limitation mechanism observed in cracker systems [29], gradient reduction of laminating fat in laminated pastries also suppresses acid and peroxide formation. However, critical distinctions emerge when comparing the present work with reduced-fat croissant research that relies on oleogel fat replacers to compensate for textural deficits. As reported by Esmaeilinezhad et al. [22], croissants reformulated with sunflower oil-based water-in-oleogels only achieved marginal declines in peroxide value despite successful fat reduction. The poor oxidative improvement stems from the high polyunsaturated fatty acid content of vegetable oil oleogel matrices, which are inherently more susceptible to free radical oxidation than dairy butter lipids, offsetting the stability gain brought by lower total fat content. In contrast, the current study only adjusts the butter dosage without introducing any plant-derived structured lipid substitutes rich in unsaturated fatty acids, eliminating additional oxidation-prone lipid fractions and achieving a more prominent reduction in both acid and peroxide indices.

Notably, the superior oxidative stability observed here is only validated under short-term fresh storage conditions. Though lower lipid loads slow initial rancidity reactions, the elevated moisture content of reduced-fat Katima creates a favorable microenvironment for moisture-mediated lipid oxidation and microbial proliferation during prolonged commercial shelf life, a confounding factor that also requires consideration in scaled industrial production as summarized in previous laminated pastry reviews.

(Note: All experiments were performed in triplicate. Data are shown as means ± standard deviation. Values with different letters in the same column differ significantly (*p* < 0.05).)

### 3.7. Sensory Evaluation Results of the Optimal Formula

The sensory evaluation results of the nutrition reduced-fat Katima produced by the optimal formula are shown in Table 7.

The individual and total sensory scores of Katima produced with the optimized formulation are summarized in Table 8. For the four sensory evaluation dimensions consistent with Table 2, the optimized sample obtained scores of (19.1 ± 0.4) for Color, (19.3 ± 0.3) for Texture, (38.2 ± 0.5) for Taste, and (18.4 ± 0.3) for Smell. The sum of these sub-index scores yielded an average total sensory score of (95.03 ± 0.39) (*n* = 3), which falls within the 95% confidence interval [94.25, 96.99] predicted by the RSM model. The high scores across all sensory attributes demonstrate that the optimized reduced-fat Katima possesses well-balanced color, texture, taste, and aroma characteristics. The strong consistency between experimental sensory results and model predictions further verifies the validity and reliability of the established response surface regression model for Katima formulation optimization.

(All data are expressed as the mean ± standard deviation (*n* = 3). Color, texture, and smell are scored out of 20 points each, while taste is scored out of 40 points.)

## 4. Conclusions and Outlook

This study took traditional Xinjiang Katima as the research object to explore the formulation optimization scheme for reduced-fat reformulation of this ethnic layered pastry, using high-gluten flour as the base raw material and butter, yeast, salt, egg liquid, and raisins as matching auxiliary materials. Single-factor experiments were first performed to clarify the separate influence of each raw material addition level on dough rheological characteristics, finished product texture, surface chromaticity, and sensory attributes. On the basis of the effective concentration intervals screened from single-factor tests, response surface methodology was adopted with comprehensive sensory scores as the core evaluation indicator to establish a quadratic regression model describing the correlation between three key variables (butter dosage, yeast dosage, and salt dosage) and overall product quality, and the model exhibited reliable fitting performance. The desirability function method was further applied to carry out multi-objective optimization to obtain the optimal compound formulation, and follow-up validation tests confirmed that the optimized reduced-fat Katima maintained satisfactory sensory performance while achieving balanced physicochemical indicators, including texture profile, surface color, moisture content, acid value, peroxide value, and total fat content, and all test indicators met the requirements of China’s national food safety standard series GB 5009 [30].

A systematic comparative analysis of macroscopic physicochemical properties, textural features, color parameters, lipid oxidation stability, and sensory quality was implemented between optimized reduced-fat Katima and traditional full-fat Katima, and all analytical discussions in this paper are strictly derived from measured experimental data. In terms of nutritional labeling specifications, this study referenced CAC/GL 23 and China’s national standard GB 28050 [31] simultaneously for standardized judgment of reduced-fat claims: the total fat content of optimized Katima was 10.77 g per 100 g, which was 37.21% lower than the 17.21 g per 100 g of the traditional control group, satisfying the domestic standard’s 25% fat reduction threshold for “reduced-fat” labeling, but failing to reach the stricter international CAC standard threshold of no more than 3 g fat per 100 g solid food for corresponding nutritional claims. It should be noted that the present research only analyzed the changes in macroscopic quality and lipid oxidation stability caused by butter reduction from the perspective of basic physicochemical detection, without supplementary microstructural and molecular characterization means such as scanning electron microscopy and Fourier-transform infrared spectroscopy, so direct evidence at the microscopic and molecular levels to interpret the regulation mechanism of fat reduction on pastry internal matrix structure cannot be provided in this work.

Given the absence of shelf-life monitoring, consumer acceptability evaluation, and human nutritional intervention trials in the current experimental design, the industrial application potential and health-related value of this optimized formulation can only be preliminarily inferred from existing physicochemical and sensory data without overstated assertions. For subsequent follow-up research, we plan to introduce SEM, FTIR, and other instrumental characterization technologies to reveal the evolution law of gluten network structure and intermolecular interaction forces under reduced-fat conditions and additionally carry out storage shelf-life tracking, consumer sensory acceptance tests, and relevant nutritional assessment experiments to supplement sufficient data support for evaluating the industrial promotion prospect and actual health benefits of reduced-fat Katima products.

## Figures and Tables

**Table 1:** Detailed steps of the Katima production process.

Operating Steps	Detailed Procedure
Flour mixing	Predetermined high-gluten flour was used as the base material. The weighed raw materials, including 100 g high-gluten flour, egg liquid, instant active dry yeast, pretreated raisins (destemmed, rinsed, and air-dried), and refined edible salt, were added to the mixing vessel sequentially. A planetary stirrer was adopted for dry blending to fully homogenize all components within the flour matrix and form a uniform powder premix. The dough hydration level was fixed at 40% (*w*/*w*, flour basis).
Mixing and cutting	Purified water was added to the premixed flour in three separate portions at a dosage of 40 g per 100 g flour, which facilitated complete water uptake by flour particles and the development of a continuous gluten network. This process imparted satisfactory elasticity and processability to the dough. After kneading, the well-blended dough was transferred to a constant-temperature incubator for primary fermentation at 32 °C with 85% relative humidity; fermentation lasted 60 min until the dough volume doubled. Fermented dough was divided into identical individual blocks with a pre-forming weight of 60 g for the subsequent laminating procedures.
Rolling, greasing, shaping, and forming	Each dough block was rolled into an initial dough sheet with a uniform thickness of 8 mm and dimensions of 30 cm (length) × 20 cm (width). A total of three consecutive three-folding cycles were repeatedly performed to construct the layered structure of Katima. Subsequently, a fixed quantity of unsalted butter was evenly spread onto each laminated dough sheet. The butter-coated dough sheets were tightly rolled into long strips and spirally coiled into neat circular blanks. Stainless steel baking trays (40 cm × 60 cm, standard size) were lightly coated with food-grade release agent to ensure non-stick performance, which facilitated complete demolding and preserved the integrity of the pastry shape after baking.
Baking and cooling	The electric oven was preheated for 30 min, with the upper heating temperature set at 180 °C and the bottom heating temperature at 160 °C. All shaped dough blanks were evenly placed on greased baking trays and baked for 15 min. Upon completion of baking, the freshly prepared Katima pastries were transferred to a cool, well-ventilated workbench and allowed to cool naturally to room temperature. After cooling, each finished Katima piece had a final weight of approximately 55–58 g. All cooled samples were immediately sealed in polyethylene plastic bags and stored under constant conditions (25 ± 1 °C, 50–55% relative humidity) for 24 h before texture profile analysis, color difference measurement, and sensory evaluation.
Replicate definition	Technical replicates: Triple measurements from one dough batch for physicochemical analysis to minimize testing error. Biological replicates: Three independently produced pastry batches for sensory evaluation to reflect manufacturing batch variation. All results are expressed as the mean ± standard deviation of the corresponding triplicates

**Table 2:** Factors and levels for RSM.

Level	Factor		
	Amount of Butter Added (A, g)	Amount of Yeast Added (B, g)	Amount of Salt Added (C, g)
−1	10	0.6	0.4
0	12.5	0.8	0.6
1	15	1	0.8

**Table 3:** Sensory indicators of the Katima.

Evaluation Index	Description	Score (Total Score 100)
Colour	Bright orange crust, uniform gloss Slightly uneven colour, partial matte surface Dark crust, obvious dullness Severely discolored, fully lackluster	15~20 10~15 5~10 Under 5
Texture	Intact shape, even thickness, clear pattern Complete shape, blurred pattern Uneven thickness, minor expansion Deformed, severe foaming voids	15~20 10~15 5~10 Under 5
Taste	Tender crumb, balanced authentic flavour Mild characteristic taste; no defects Dry/hard crumb, faint bland flavour Dense texture, distinct rancid/burnt off-taste	30~40 20~30 10~20 Under 10
Smell	Rich typical pastry aroma Weak intrinsic scent, no off-odors Faint volatile flavour Obvious rancid or burnt odor	15~20 10~15 5~10 Under 5

**Table 4:** Response surface test design and results.

Order Number	A-Amount of Butter Added (g)	B-Yeast Addition Amount (g)	C-Amount of Salt Added (g)	Sensory Rating-Y
1	15	0.8	0.8	82.3
2	10	0.8	0.8	83.2
3	12.5	0.8	0.6	95.3
4	15	0.6	0.6	84
5	12.5	0.6	0.4	91.2
6	12.5	0.8	0.6	95
7	15	1	0.6	88.2
8	12.5	0.8	0.6	96
9	10	0.6	0.6	86.4
10	12.5	0.8	0.6	95.3
11	12.5	0.6	0.8	82
12	10	1	0.6	81
13	15	0.8	0.4	86.8
14	12.5	1	0.4	84.5
15	10	0.8	0.4	83.6
16	12.5	0.8	0.6	95.5
17	12.5	1	0.8	85.6

**Table 5:** Analysis of Variance Table.

Variance Source	Quadratic Sum	Degrees of Freedom	Mean Square	F	*p*	Significance
model	480.95	9	53.44	122.23	<0.0001	**
A-Amount of butter added	6.30	1	6.30	14.41	0.0067	**
B-yeast addition amount	2.31	1	2.31	5.29	0.0551	
C-Amount of salt added	21.13	1	21.13	48.32	0.0002	**
AB	23.04	1	23.04	52.70	0.0002	**
AC	4.20	1	4.20	9.61	0.0173	*
BC	26.52	1	26.52	60.66	0.0001	**
A^2^	161.07	1	161.07	368.40	<0.0001	**
B^2^	79.13	1	79.13	180.98	<0.0001	**
C^2^	116.50	1	116.50	266.45	<0.0001	**
Residual	3.06	7	0.4372			
Lack of Fit	2.51	3	0.8375	6.11	0.0564	
Pure error	0.5480	4	0.1370			
Total deviation	484.01	16				
Correlation coefficient	R^2^ = 0.9937, AdjR^2^ = 0.9855, PredR^2^ = 0.9152					

Note: Values marked with different lowercase letters in the same column differ significantly (*p* < 0.05); * *p* < 0.05, ** *p* < 0.01.

**Table 6:** Comparison of Key Differences Between Reduced-Fat Katima and Traditional Katima.

Product	Characteristic																		
	Hardness	Springiness	Adhesion	Chewiness	Adhesion	Cohesiveness	r/cm	H/cm	Volume/cm^3^	Mass/g	Crumb Density/(g/cm^3^)	Water/%	Fat/%	Peroxide Number (meq O_2_/kg)	Acid Value (mgKOH/g)	L*	a*	b*	ΔE
reduced-fat Katima	6.80 ± 1.24 ^a^	5.14 ± 0.71 ^a^	3.22 ± 1.82 ^a^	22 ± 4.95 ^a^	0.35 ± 0.32 ^a^	0.47 ± 0.25 ^a^	2.4 ± 0.36 ^a^	5.87 ± 0.71 ^a^	109.202 ± 37.51 ^b^	59.23 ± 10.74 ^a^	0.5741 ± 0.1360 ^a^	9.627 ± 0.331 ^a^	10.77 ± 0.200 ^a^	275.97 ± 0.037 ^a^	0.111 ± 0.004 ^a^	58.51 ± 1.49 ^a^	8.98 ± 3.87 ^a^	34.94 ± 6.02 ^a^	14.65
Traditional Katima	2.59 ± 1.41 ^b^	1.92 ± 0.90 ^b^	1.54 ± 0.70 ^b^	3.8 ± 2.26 ^b^	2.32 ± 1.05 ^b^	0.23 ± 0.06 ^b^	2.43 ± 0.06 ^a^	6.27 ± 1.57 ^a^	115.905 ± 25.16 ^a^	60.797 ± 1.135 ^a^	0.5369 ± 0.0910 ^a^	7.95 ± 0.84 ^b^	17.21 ± 0.523 ^b^	316.34 ± 0.061 ^b^	0.132 ± 0.011 ^b^	68.13 ± 2.22 ^b^	0.65 ± 2.38 ^b^	27.69 ± 5.99 ^b^	

Notes: Values are expressed as mean ± standard deviation (*n* = 3). Different lowercase superscript letters (a, b) within the same column indicate significant differences at the level of *p* < 0.05 between reduced-fat Katima and traditional Katima according to LSD multiple range test. Identical lowercase superscript letters in one column represent no statistically significant difference (*p* > 0.05).

**Table 7:** Sensory evaluation results of optimized reduced-fat Katima.

Evaluating Indicator	Evaluation Results
Color	Bright orange shell with an even sheen and no signs of scorching.
Taste	It has a mild flavor with notes of butter and raisins, and is moderately sweet.
Smell	It has aromas of wheat, a mild buttery and milky scent, and subtle raisin notes; the aroma is pleasant and mellow, with no harsh or unpleasant odors.
Texture	Soft and fluffy texture, no roughness, and non-sticky. Complete shape with uniform size; the cross-sectional structure is loose and porous, and free of impurities

**Table 8:** Individual and total sensory scores of optimized reduced-fat Katima.

Sample	Color	Texture	Taste	Smell	Total Sensory Score
reduced-fat Katima	19.1 ± 0.4	19.3 ± 0.3	38.2 ± 0.5	18.4 ± 0.3	95.03 ± 0.39

## Data Availability

The raw experimental data generated in this study are not publicly available at present, since these datasets will be further analyzed and extended for follow-up in-depth research on reduced-fat Katima. The corresponding data will be shared openly upon the completion and publication of our subsequent related research.

## Abbreviations

Abbreviation	Full Name
RSM	Response Surface Methodology
ALCs	Amylose-lipid complexes
ANOVA	Analysis of Variance
BBD	Box-Behnken Design
CV	Coefficient of Variation
LSD	Least Significant Difference
HS	Honest Significant Difference
CIE	Commission Internationale de l’Eclairage
ISO	International Organization for Standardization
CAC/GL	Codex Alimentarius Commission Guidelines
SPSS	Statistical Package for the Social Sciences
SEM	Scanning Electron Microscope
FTIR	Fourier Transform Infrared Spectroscopy
